# Association and Effects of Trauma, Displacement, and Illicit Drug Use on Psychiatric Illnesses in Khyber Pakhtunkhwa, Pakistan

**DOI:** 10.7759/cureus.22079

**Published:** 2022-02-10

**Authors:** Muhammad Noman K Wazir, Kaneez Fatima, Hooria R Ahmad, Susan Kakakhel, Nowsher Yousaf, Fakhria Wahid

**Affiliations:** 1 Psychiatry, Northwest General Hospital and Research Centre, Peshawar, PAK; 2 Psychology, Islamia College Peshawar, Peshawar, PAK; 3 Psychiatry, Rehman Medical Institute, Peshawar, PAK; 4 Physiology, Northwest School of Medicine, Peshawar, PAK; 5 Occupational Health, Northwest General Hospital and Research Centre, Peshawar, PAK

**Keywords:** tribal area, northwest pakistan, afghanistan, psychological issues, psychiatric illnesses, trauma, drug use, war, displacement, refugees

## Abstract

Methodology

Data were accumulated from all patients seen in outpatient clinics from October to December 2020. No inpatients or community samples were included in the research. Verbal consent and approval from the local ethical committee were obtained beforehand. ICD-10 diagnostic criteria were used for all psychiatric diagnoses.

Results

A total of 500 patients were seen with a gender distribution of 51% females and 49% males. Patients aged 18-65 years comprised 79% of this sample; 52% had no formal education. A total of 43% lived in settled areas; 37% were from Afghanistan; 13% from tribal areas, and 7% were from the Swat region. Thirty percent had no medical history, and 40% reported no prior contact with psychiatric service. Anxiety spectrum disorders* *were more prevalent in females, and psychotic and drug-related illnesses were more prevalent in males.* *More than half the patients seen were considered fit for psychotherapy referral but could not engage because of the lack of such services near their homes. The significance of the results obtained was assessed using the Chi-squared test, using SPSS v.22. A p-value of less than 0.05 was considered significant.

Conclusion

Almost 40% of patients were admitted due to some form of trauma history, predominantly terrorism-related, displacement, and other losses. Two in three people reported current or past drug use, with tetrahydrocannabinol (THC) being the most commonly used drug, followed by sedatives, opioids/pain relief medications, amphetamines, or methamphetamine (ICE), and others (e.g., alcohol). There was a significant rise in drug use/trauma history in the tribal areas, Afghanistan, and Swat region compared to the local population of settled areas. Common psychiatric illnesses were as expected in the sample studied.

## Introduction

Khyber Pakhtunkhwa (KP), formally known as the North-West Frontier Province, is one of the four administrative provinces of Pakistan, located in the country's northwestern region along the international border with Afghanistan. KP is the third largest province of Pakistan by the size of both the population and economy. According to 2011 estimates, the province has an estimated population of about 27.5 million (11.9% of Pakistan's total population), of whom 52% are males, and 48% are females. Around 1.5 million Afghan refugees also stay in the province.

The tribal area, which is now part of the settled area (areas that have an established setup, run by the government and local bodies), is spread over 27,220 sq km and has a population of 3,764,000. It comprises seven tribal agencies and six frontier regions. The tribal agencies are Bajaur, Mohmand, Khyber, Orakzai, Kurram, North Waziristan, and South Waziristan.

Over the last two decades or so, KP, the associated tribal belt, and Afghanistan have been major centers of militancy, terrorism, trauma, and the associated displacement of refugees, internally in Pakistan and Afghanistan.

This study aimed to find out the association of a history of trauma and drug use with psychiatric illnesses and to check the availability and willingness of the population to engage in psychological therapies for these and other psychological issues.

Trauma can occur after one experiences an event that hurts physically or emotionally. Trauma has enduring effects on mental, physical, and emotional wellbeing [1]. Experiencing abuse or other trauma can put people at risk of developing various mental health conditions. Trauma includes dangerous, frightening, or highly stressful situations or events, such as sexual assault, war, an accident, natural disaster, the sudden or violent death of a close loved one, or a serious physical health problem.

It has been shown that the long-term effects of abuse or trauma include severe anxiety, stress, post-traumatic stress disorder (PTSD), schizophrenia, bipolar illness, alcohol or drug abuse, depression, eating disorders, self-injury, suicide, and other mental illnesses [2]. In addition, abundant evidence has shown that childhood trauma compromises neural structure and function, rendering an individual predisposed to later cognitive deficits and psychiatric illnesses [3-4].

For the most part, the link between trauma exposure and substance abuse has been well-established. For example, in the National Survey of Adolescents, teens who experienced physical or sexual abuse or assault were three times more likely to report past or current substance use disorders (SUDs) than those without a history of trauma [5]. In addition, SUDs have high comorbidity with PTSD and other mood-related psychopathologies [6].

It has been suggested that people with mental illness tend to be or become reliant on substances than individuals who do not have mental disorders [7]. Conversely, individuals who abuse substances are more likely to develop or suffer from mental illnesses than those who do not abuse substances [8]. Thus, when mental illness and problematic substance use or abuse co-occur, the resulting problems are often intensified and complex [9].

It is important to understand that SUDs are generally divided into two categories: substance dependence and substance abuse. Substance dependence is generally considered more severe than substance abuse. The participants of our study belonged to the latter category.

## Materials and methods

A total of 500 participants, aged between 12 and 80 years, who presented or were referred to our psychiatric practice in October, November, and December 2020, consented to be enrolled in this study. Unfortunately, patients admitted to the general hospital were not included in the sample because of a lack of inpatient psychiatric facilities.

The clinical setting was Northwest General Hospital and Research Centre, a 350-bed private hospital catering to the people of KP, Pakistan, and Afghanistan.

Information such as demographics (e.g., age, gender, educational statutes, address) was collected and tabulated (using predetermined tables). As we had set out to find and confirm the association between a history of trauma and drug use on one’s mental health, we gathered further details about these patients, including any history of trauma (direct or indirect), current or past drug use, the type of drug used, and any psychiatric and medical history. Finally, all patients were assessed and given a psychiatric diagnosis based on the International Classification of Diseases, 10th revision (ICD-10) diagnostic criteria.

Lastly, we gathered information from every participant about their perceptions on any form of available psychological therapy (counseling, cognitive-behavioral therapy (CBT), psychodynamic and psychoanalytic therapy, motivational enhancement therapy, and so on), their willingness to engage in such therapies, and the availability of such services in their area.

The authors confirm that all procedures contributing to this work conform with the ethical standards of the relevant national and institutional committees on human experimentation and with the Helsinki Declaration of 1975, as revised in 2008. In addition, the ethical committee of Northwest General Hospital and Research Center approved all procedures involving human subjects. Verbal consent was recorded from all participants included in the study.

The significance of the results obtained was assessed using the Chi-squared test, using IBM SPSS version 22. A p-value of <0.05 was considered significant.

## Results

The total number of patients included in this study was 500. Patients admitted to the hospital were not included in the study because of a lack of inpatient beds. The sample consisted of 51% females and 49% males. Those aged from 18 to 65 years comprised 79% of the study sample, <18 years comprised 11%, and those >65 years comprised 10%. A total of 52% of the subjects were uneducated, and 48% were educated or receiving an education. A total of 43% of them lived locally (in settled areas), 37% were from Afghanistan, 13% from the tribal areas, and 7% from the Swat region. Of the subjects, 31% had no medical history, and 40% reported no prior contact with psychiatric services (Table 1).

**Table 1:** Demographics. TA: Tribal areas.

Distribution by sex	Females: 255			Males: 245			51%:49%
Distribution by education	Educated: 239			Uneducated: 261			48%:52%
Distribution by age	<18 years: 56		18-65 years: 396		>65 years: 48		Mean age: 32 years
Distribution by address	Local: 215	TA: 66		Afghanistan: 184		Swat: 36	43%:13%:37%:7%
History of trauma	Yes: 179			No: 321			36%:64%
Medical history	Yes: 343			No: 157			69%:31%
Psychiatric history	Yes: 301			No: 199			60%:40%

Almost 40% of the patients were admitted due to some form of trauma history, predominantly terrorism-related, displacement, and other losses. Two in three people reported some form of drug use (either current or a history of use), with tetrahydrocannabinol (THC) being the most commonly used drug, followed by sedatives, opioids, or synthetic opioid analgesics, amphetamines or ICE, and others (e.g., alcohol). ICE and pain medicines were significantly (p-value <0.001) associated with drug-induced psychosis and panic attacks, respectively. Drug use was also significantly (p-value <0.015) related to past psychiatric illness in tribal areas. Furthermore, a significant relationship was observed between past psychiatric history and THC, as well as ICE use.

Illicit drug use was the most prevalent in patients from the tribal areas (34%), followed by those from Afghanistan (27%), the Swat region (21%), and the local population (18%). A total of 36% of the patients were admitted due to some history of trauma (n = 178); 68% of them also had current or past drug use. Compared to the local population, a history of trauma was more common in the non-local population, particularly from the Swat region (p-value <0.03). Moreover, a history of trauma was linked to past psychiatric illness in Afghanistan and tribal areas.

All patients were screened for referral to available psychological services (government provided/free or private). Of those, 8% were not deemed appropriate for a referral. Successful referrals were made for 13% of the patients; 24% were referred but refused engagement with such services. Unfortunately, and as expected, 55% were deemed appropriate and in need of psychological interventions but could not engage with therapy because of either non-availability of services or they had no access to it. Although the local population was not keen on seeking therapy, people from Afghanistan and tribal areas faced accessibility issues (p-value <0.0002) (Table 2).

**Table 2:** Drug use by types and population, and the psychotherapy outcome. THC: tetrahydrocannabinol/cannabis; ICE: methamphetamine; TA: Tribal areas.

Drug use (n = 122)	THC: 56	Sedatives: 22	Opioids/Pain relief meds: 18	ICE: 14	Others: 12
Drug use by population (n = 122)	Local: 48	TA: 19	Afghanistan: 47	Swat: 8	Percentage: 22.4:28.7:25.5:22.2
Psychotherapy referral (n = 500)	Not indicated: 73	No access: 250	Referral done: 30	Not keen: 147	Percentage: 14.6:50:6:29.4

The most prevalent mental illnesses were noted to be mixed anxiety and depression (MAD), generalized anxiety disorder (GAD), depression, panic attacks, dissociative states, obsessive-compulsive disorder (OCD), bipolar affective disorder (BPAD), schizophrenia, developmental disorders, dementia, drug-induced psychosis, social phobia, and others.

Furthermore, illnesses more prevalent in males included GAD, schizophrenia, BPAD, dementia, developmental disorders, social phobia, and drug-induced psychosis. On the other hand, females had a higher percentage of MAD, depression, panic attacks, PTSD, acute stress reactions, and dissociative states.

**Figure 1:**
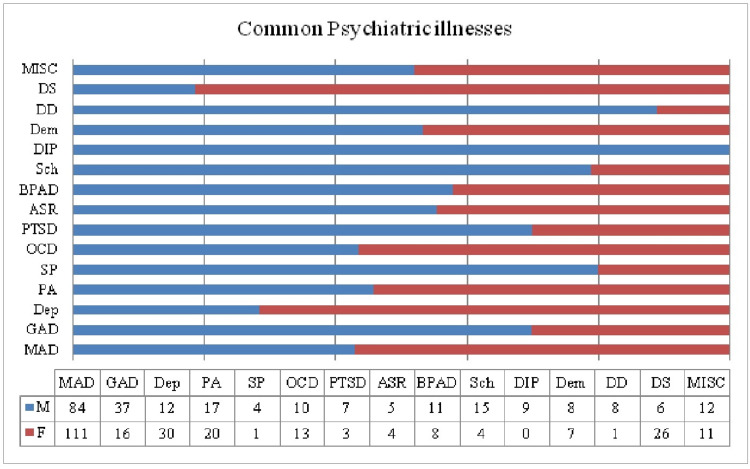
Prevalence of common psychiatric illnesses. MAD: Mixed anxiety and depression; GAD: Generalized anxiety disorder; Dep: Depression; PA: Panic attacks; SP: Social phobia; OCD: Obsessive-compulsive disorder; PTSD: Post-traumatic stress disorder; ASR: Acute stress reaction; BPAD: Bipolar affective disorder; Sch: Schizophrenia; DIP: Drug-induced psychosis; Dem: Dementia; DD: Developmental disorders; DS: Dissociative syndrome; MISC: Miscellaneous.

Anxiety spectrum disorders were more prevalent in females, and psychotic and drug-related illnesses were more prevalent in males. Schizophrenia, drug-induced psychosis, and BPAD were significantly associated with drug use (p-value <0.0002) (Figure 1).

## Discussion

The associations between increased incidence of drug use and psychiatric illness, trauma and drug use, and trauma plus drug use and psychiatric illnesses are well established and backed by a considerable volume of research. In addition, there is a statistically significant association between the history of armed conflict, violence, and the presence of mental illnesses, particularly depression, somatization disorder, and alcohol abuse [10].

This study aimed to analyze the same associations in the local population and those affected by years of unrest, war and terrorism, and the associated displacement. This study focused on similar incidences in this population group, limited not only to Pakistan but also to Afghanistan, affected by similar circumstances.

Our study confirmed these objectives and was able to look at an adequate sample size composed of people from all these affected areas.

As noted in our study, certain illnesses were more prevalent in females than in males. These included depression and anxiety-related disorders, such as panic attacks, GAD, and dissociative syndrome. The male population presented mainly with psychotic illnesses. The most common drug use in males was THC, opioids, and ICE compared to sleeping tablets and opioid pain relief medications in females. The main reason for this discrepancy in the preferred drug of abuse between the two genders is their access to them.

People from the tribal areas, Afghanistan, and the Swat region, showed a higher incidence of trauma, drug use, and psychiatric illnesses than patients from the settled areas. This is consistent with findings from previous research conducted on different global populations affected by similar circumstances [11-15]. Many factors identified in research studies are modifiable and, thus, interventions aimed at prevention often result in reductions in both SUDs and mental illnesses [15].

Stress remains a proven risk factor for mental disorders and, therefore, provides one possible universal neurobiological relationship between substance abuse and psychiatric illnesses [16-18]. Stress responses, mediated through the HPA axis, influences our brain circuits that control motivation. Elevated stress levels lower activity in the prefrontal cortex and augment responsiveness in the striatum, leading to lowered behavioral control and increased impulsivity [19]. It has been shown that early life stress and chronic stress cause long-term changes in the HPA axis, affecting limbic brain circuits involved in learning, motivation, and adaptation. These are impaired in those with SUDs and other mental illnesses [20-23].

In conjunction with the above, the dopamine pathways are also implicated in how stress increases our susceptibility to SUDs. Secondarily, HPA axis hyperactivity alters dopamine signaling and augments the reinforcing properties of illicit drugs [16,18-19]. As a result, psychological therapies, such as mindfulness, can be advantageous in reducing anxiety, depression, and substance use [23].

Substance abuse can be understood as numbing or erasing the pain caused by trauma [11]. The abuse of pain relief medications in the female population is of particular importance, possibly to relieve all the somatic complaints and associated pains or aches.

Besides the easy availability of these drugs of abuse, certain intrinsic predisposing factors were considered. For example, in the Pukhtoon/Pashtun (majority of our studied population) way of life, THC usage is not considered bad; in fact, it is even promoted at gatherings and meetups. Smoking and Naswar (chewed tobacco) usage: the true gateway drugs are used as a coming of age drug and cement one's transition into adulthood in the rural areas.

Similarly, opioid is not home-grown but has been imported from our neighbors, Afghanistan. Again, poor legislation and easy availability have been a menace to our society for the last few decades. Kabul remains the opium capital of the world, and Afghanistan is the source of 75% of the world's heroin. Most of it is trafficked through Pakistan on its way to money-spinning foreign markets [24].

Furthermore, the availability of better rehabilitation services and changing the general perceptions of society through education and the use of all available platforms are imperative. However, as shown in our study, more than half of the subjects deemed in need of and fit for psychological input did not want to engage in it.

## Conclusions

Our study focused on the areas and the population affected by trauma in the form of years of civil unrest and hence showed a greater incidence of not only the common psychiatric illnesses but also different forms of illicit drug use. THC usage was more common in men, followed by opioid use and ICE. Sedatives and analgesics were found to be abused by women more.

Easy access to these illicit substances, the local culture or traditions, and poor control and legislation regarding the use of prescribed medications also play a role in promoting and maintaining such maladaptive ways of coping.

Stigma, a lack of education, limited understanding of mental illness and psychological issues, financial restraints, and lack of provision and feasibility of counseling services were identified as the primary reasons why people are unwilling to engage in such treatments; therefore, these need to be also addressed.

The government, public and private sectors, all need to play their roles in improving and spreading awareness regarding mental illnesses and drug abuse.
